# *Operando* Electrochemical and Optical Characterization of the Meniscus of Scanning Electrochemical Cell Microscopy (SECCM) Probes

**DOI:** 10.1021/acselectrochem.4c00029

**Published:** 2024-10-07

**Authors:** Dimitrios Valavanis, Paolo Ciocci, Ian J. McPherson, Gabriel N. Meloni, Jean-François Lemineur, Frédéric Kanoufi, Patrick R. Unwin

**Affiliations:** † Department of Chemistry, University of Warwick, Coventry CV4 7AL, United Kingdom; ‡ Université Paris Cité, ITODYS, CNRS, F-75013 Paris, France; § Institute of Catalysis Research and Technology, Karlsruhe Institute of Technology, 76344 Eggenstein-Leopoldshafen, Germany; ∥ Department of Chemistry, Loughborough University, Loughborough LE11 3TU, United Kingdom; ⊥ Institute of Chemistry, Department of Chemistry, University of São Paulo, São Paulo, SP 05508-000, Brazil

**Keywords:** opto-electrochemistry, scanning electrochemical cell microscopy, nanopipette meniscus, nanodroplet

## Abstract

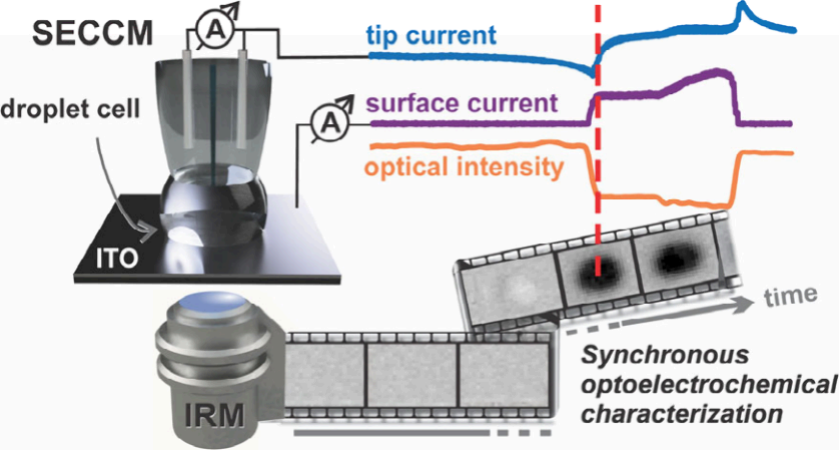

We present a thorough description of the scanning electrochemical cell microscopy (SECCM) meniscus probe, in operation, by combining dual-channel SECCM measurements with *in situ* interference reflection microscopy (IRM). SECCM is a pipette-based nanoscale characterization tool with an unparalleled capacity for mapping the electrochemical activity of material surfaces, with high precision and at high throughput. In hopping mode, it operates by bringing the electrolyte meniscus, at the scanned pipette tip, in contact with the sample, restricting the probed area each time to a separate, newly wetted site, and forming a small-scale reactor. Each contact area can normally be imaged post-experiment, to inform on the wetted area stability and enable quantitative data interpretation (e.g., to calculate current density). However, the description of meniscus behavior during measurements would be beneficial. Herein, we utilize semi-transparent electrode substrates, to enable the direct optical observation, by IRM, of the meniscus status, with high spatial and temporal resolution, and synchronously to SECCM operation. The surface-sensitive optical method allows us to accurately capture the nature of the miniature electrochemical cell during all phases of the experiment—during approach, meniscus contact, wetting, and pipette withdrawal—and to follow subtle changes while in contact with the electrode substrate. Through the use of a dual-channel probe, we are able to monitor both the ionic current across the meniscus, between quasi-reference counter electrodes (QRCEs) under bias, and between the working electrode surface and the QRCEs. Correlating these electrochemical data and *operando* optical information via the hybrid SECCM-IRM approach aids the design of experimental protocols, streamlines the interpretation of results, and paints a comprehensive picture of meniscus wetting behavior.

## Introduction

Scanning electrochemical probe microscopy (SEPM) experimental methods are analytical measurement techniques,^1,2^ developed to interrogate interfacial processes locally, at small scales, across a range of materials^3^ or biological samples.^4,5^ The probe is typically moved around the area of interest in a scanning array fashion, following an automated experimental protocol, inducing reactions and sensing the local physicochemical environment. The unveiling of spatially heterogeneous electrochemical activity, as demonstrated by such measurements, may inform *inter alia* on the design of electrocatalysts^2^ and battery electrode materials,^3^ the advancement of protective coatings,^6^ or the effect of pharmaceuticals on tissues and cells.^7^ Among SEPM methods, scanning electrochemical cell microscopy (SECCM)^8^ is unique in its capacity for direct probing of a well-defined sample area, at micrometre or nanometre scales, in a high-throughput manner.^9,10^ It has the capacity to inspect local topography and electrochemical activity independently and simultaneously,^11^ while showing versatility towards a range of different interfaces and experimental conditions. It has been applied to study the performance of electrocatalysts^12,13^ and battery materials,^14,15^ corrosion phenomena,^16,17^ proton permeation through two-dimensional membranes,^18^ metal electrodeposition,^19−21^ and many other systems.

SECCM utilizes the electrolyte meniscus formed at the tip of a moving pipette probe to interrogate the sample surface, only within the confines of the area upon which it lands and wets. The method represents a significant advance on the scanning microdroplet technique,^22^ with improvements in the experimental configuration, the adoption of a high-precision control unit, and a thorough understanding of the underpinning theory that influences measurements.^23^ In its modern format, SECCM is able to capture surface features of different scales,^24,25^ with facile access to the nanoscale.^11,26^ The researcher can image the electrochemical response of the sample under specific atmospheric conditions, temperature control, or light illumination.^27−29^ The sample can then be taken for co-located *ex situ* characterization (e.g., electron microscopy, atomic force microscopy, or Raman spectroscopy), in order to ultimately construct a multi-microscopic representation of the surface and uncover structure–activity relations.^30−32^ The simple, axisymmetric geometry of the pipette–substrate system facilitates its simulation by finite element method multiphysics models^33^ or its description by simple analytical formulations.^34^ SECCM has further been proven as a tool for synthesis in confinement.^35,36^ One may use scanning array protocols for repeatedly landing a freshly-formed electrolyte meniscus, each time at a new, untouched sample spot, and forming a deposit via electrochemical control of the miniature reactor.^21^ A wide parameter space can be explored, through tens or hundreds of independent tests, for process optimization in a short timeframe.^19^

More recently, the easily-customizable SECCM experimental configuration has been coupled with *in situ* optical microscopy,^37−39^ to track electrochemically-induced phase changes at the electrode–electrolyte interface, in real time. Semi-transparent and conductive substrates (e.g., indium tin oxide-coated, or gold-coated, glass) are utilized as the working electrode, secured over the objective lens of an inverted microscope. Tuning of substrate and illumination characteristics enables the use of a surface-sensitive, interference-based optical microscopy technique (interference reflection microscopy, IRM), and the unprecedented visualization of the SECCM meniscus.^38,39^

Optical observation of the sample was a feature of early SECCM experimental setups,^40^ aiding the targeting of areas of interest in the sample and the post-experiment characterization. The achievement of higher magnifications, through the use of an inverted optical microscope, has further improved the ability of SECCM to target particular features of a surface, such as particles.^37^ The IRM configuration advances optical visualization further, because it is particularly sensitive to local refractive index variations, allowing the detection of features below the diffraction limit, and with high temporal resolution.^39^ It also makes it possible to monitor the extent of the meniscus cell at each individual landing, while the chosen electrochemical method is applied. This sheds light onto a fine point of discussion, regarding the SECCM meniscus cell stability during measurements — notably on structurally heterogeneous surfaces — and related influence on data reliability and interpretation.^41^

Determination of the sample area that the SECCM meniscus wets (active electrode) has been typically carried out post-experiment via a range of *ex situ* microscopy techniques.^31^ It is usually established that the wetted area is approximately the same as the pipette tip opening,^8,30^ and that has been proven *in situ*, e.g., by scanning over a sharp feature where there is an abrupt change in surface electrochemical activity.^35,42^ However, there are certain (electrolyte or surface) systems and (ambient) conditions where that rule does not apply, and excessive or irregular wetting, which can also be driven by the electrochemical reaction, may occur.^43,44^ In that case, some mitigations can be employed, e.g., the use of an oil layer over the surface to reduce wetting,^45,46^ or limiting landing (stationary) time and applied overpotential.

**Figure 1:**
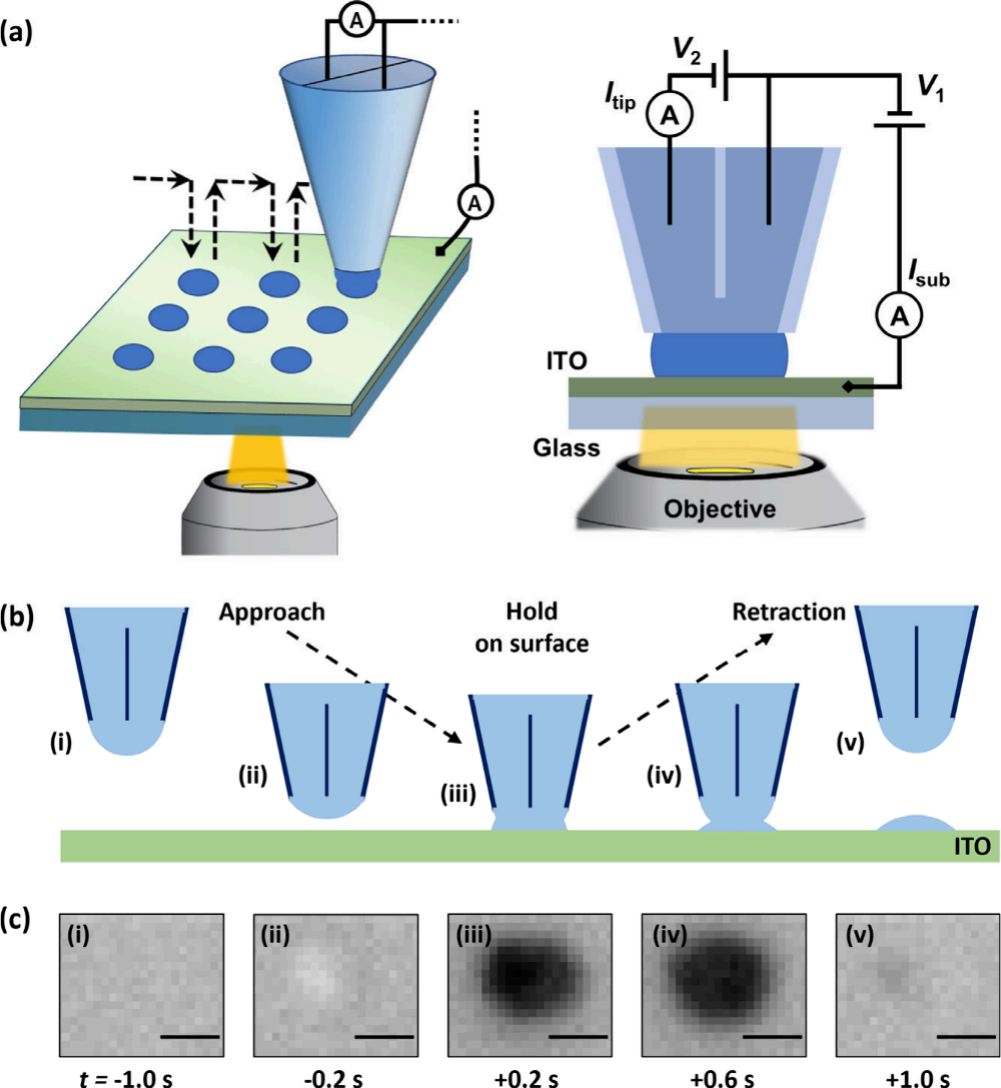
(a) Schematic of the hopping scan array protocol and the double-channel SECCM pipette tip and meniscus in contact with the ITO substrate. The optical interference effect is achieved when light coming through the objective passes through successive layers of glass, ITO, and the electrolyte. (b) Schematic, showing the protocol in which the meniscus is brought into contact with, and withdrawn from, the ITO surface (at one spot). (c) IRM snapshots, from a single SECCM hop: (i) far from the surface (−1.0 s from end of approach); (ii) close to the surface, while the feature’s intensity oscillates (−0.2 s); (iii) shortly after contact (set as time zero), with the landing spot fully wetted (+0.2 s); (iv) during retraction, while still in meniscus contact (+0.6 s); (v) after detachment, with some electrolyte residue remaining on the surface (+1.0 s). Scale bars represent 500 nm. The experiment used a ca. 500 nm tip diameter double-channel pipette with 50 mM KCl electrolyte. *V*_1_ = −750 mV, *V*_2_ = 50 mV. Effective potential applied to WE was +725 mV vs. Ag/AgCl. Tip approach and retraction rate both 1 μm s^–1^.

Studies of droplet behavior^47,48^ under electrochemical operation,^44^ or electrowetting,^49^ including some that utilize micropipette manipulation,^50,51^ are of relevance in the effort to ascertain the meniscus status, and tune its characteristics if necessary. Herein, we employ the double-channel SECCM variant,^8,23^ using theta pipette probes with a vertical septum and two separate half-cone compartments. This configuration allows for two distinct current signals to be monitored: one at the substrate (electrode surface), and one (the ionic current) between the two channels (see Figure 1 and the following sections for a detailed description of the experimental configuration).^8^ The ionic current between the two channels, under bias, is sensitive to meniscus morphology, and its characteristic current–time transients can be correlated with the *in situ* optical information, with a view to accurately describe the meniscus status.

## Experimental Section

### Reagents and Materials

Potassium chloride (KCl, ≥99%, Sigma-Alrich, UK) and 1,1′-ferrocenedimethanol (FcDM, 97%, Sigma) were used as supplied by the manufacturer. Solutions were prepared with ultrapure deionized water (PURELAB Chorus, ELGA, UK). Indium tin oxide (ITO) coated coverslips (20 mm by 20 mm, 70 nm nominal coating thickness, SPI Supplies, USA) served as the IRM substrate and were also connected as the SECCM working electrode (WE). After being cleaned by sonication in isopropyl alcohol and subsequently in deionized water, a coverslip was secured to a 3D-printed sample holder (disc with ca. 15 mm aperture, polylactic acid material).^39^ A tinned copper insulated wire was attached to the top (ITO coating) side with silver conductive paint (RS Components, UK) and the connection covered with epoxy adhesive (Araldite, UK).

Chloridized silver wires (0.125 mm diameter, annealed, 99.99%, Goodfellow, UK) were used as quasi-reference counter electrodes (QRCEs). All potentials reported herein are versus this QRCE, which has been shown to be stable over long experimental run times.^52^ A double-channel theta pipette, loaded with electrolyte solution, acted as the mobile probe for all experiments. 50 mM KCl was used initially, but in further experiments an electroactive analyte was needed, and a solution of 0.5 mM FcDM in 25 mM KCl was chosen.

Two different sizes (end aperture) of double-channel pipette probes were used: ca. 500 nm diameter pipettes made from 1.2 mm outer diameter (O.D.), 0.9 mm inner diameter (I.D.) quartz theta capillary tubes (Friedrich & Dimmock Inc., USA); and ca. 2 μm diameter pipettes from 1.5 mm O.D., 0.87 mm I.D. borosilicate theta capillary tubes (Harvard Apparatus, USA); both fabricated using a laser pipette puller (P-2000, Sutter Instrument, USA). Micropipette diameters were measured using an optical microscope before the experiments, while nanopipettes were routinely visualized using electron microscopy.^53^ Both channels of the prepared pipettes were filled with electrolyte solution, using a MicroFil syringe (World Precision Instruments Inc., USA), and a QRCE was inserted into each channel.

### SECCM-IRM Instrumentation

An inverted optical microscope (DMI4000B, Leica, Germany) was utilized as the base for the custom-built SECCM-IRM workstation.^38,39^ The blue channel LED (peak emission at wavelength λ = 470 nm, bandwidth FWHM of 19 nm) from a multi-LED light source (Niji, Bluebox Optics, UK) illuminated the coverslip through a 20× (N Plan, numerical aperture — NA — of 0.35, Leica) or a 63× oil immersion objective lens (HCX Plan Apochromatic, NA = 1.4, Leica). An additional 1.6× magnification was provided from the microscope tube lens, to attain up to 100× effective magnification. IRM images were recorded with the microscope camera (C11440-42U30, CMOS sensor, pixel size of 6.5 μm by 6.5 μm, Hamamatsu Photonics, Japan), through the MicroManager^54^ image acquisition and microscopy automation software (release 1.4.22, open source), at acquisition rates up to 330 frames per second (FPS).

The ITO substrate (WE) was mounted onto a piezoelectric positioning stage with a center aperture (Nano-Bio300 stage and Nano-Drive controller, Mad City Labs, USA), used for lateral movement in the *XY* plane, over the microscope objective. The pipette probe, loaded with the electrolyte and the QRCEs, was attached to a single axis piezoelectric actuator (P-753.3 actuator and E-665 controller, Physik Instrumente, Germany), used for moving the SECCM probe along the *Z* axis — along the optical axis and perpendicular to the WE surface. The SECCM probe was initially positioned above the substrate using coarse lateral micropositioners (M-461-XYZ-M, Newport, USA), and subsequently lowered into the near-surface position using a stepper motor (8303 Picomotor Actuator, Newport), while monitored with the lower magnification objective.

A small potential value (typically 50 mV) was applied between the two QRCEs, and the ionic current between the two channels was utilized to monitor the SECCM meniscus characteristics during the experiment.^8^ Additionally, the QRCEs were floated against the WE, which was held at ground. Electrochemical measurements were performed using custom-built current followers, sampled every 132 μs. In the event of the current value deviating from the baseline by a user-defined threshold (the noise amplitude being considered), the approach was then halted. The system was placed within a Faraday cage, lined with thermal isolation panels. The cage was situated on an active dampening table with pneumatic isolators (ISBIO-2 with Gimbal Piston, TMC, USA). Probe and substrate positioning, electrode potential control, and current sampling were managed by an FPGA card (USB-7856R OEM, National Instruments, USA), in turn governed by a LabVIEW (release 2019, National Instruments) user interface running the Warwick Electrochemical Scanning Probe Microscopy (WEC-SPM)^55^ software. The camera exposure output trigger signal was also recorded with the WEC-SPM platform, allowing for a common time frame for electrochemical and optical data.

Processing of electrochemical data and images was carried out using MATLAB (release R2019b, The MathWorks, Inc., USA) and Fiji-ImageJ^56^ (release 1.53, open source) software packages. Optical simulations were performed using Python.

## Results and Discussion

**Figure 2:**
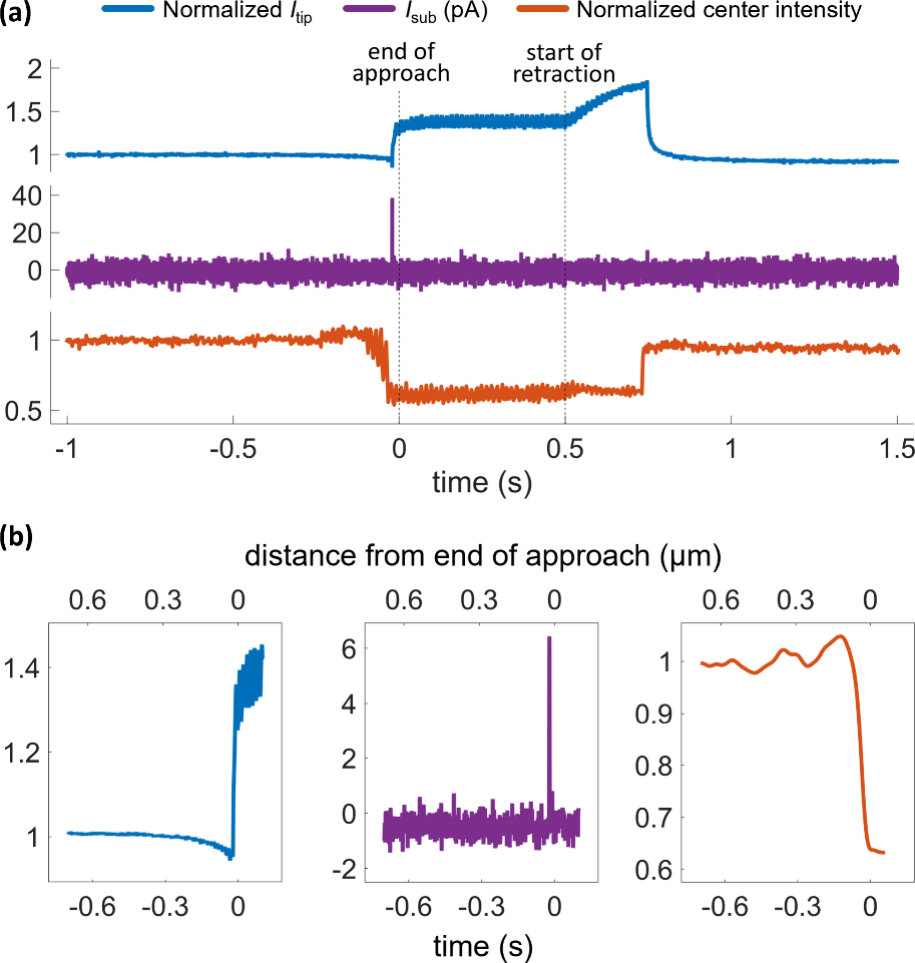
(a) Normalized tip current, *I*_tip_ (blue); raw substrate current, *I*_sub_ (purple); and normalized pixel intensity at the center of the landing spot (orange) for a single, complete SECCM hop. Vertical dotted lines denote the end of approach (approach threshold reached) and the start of retraction. Time zero refers to the end of approach. (b) Sections of the traces in (a), averaged in time by using a Gaussian filter and a 25-sample window. Raw, non-normalized *I*_tip_ traces are displayed in the SI, Figure S3. The experiment used a ca. 500 nm tip diameter double-channel pipette with 50 mM KCl electrolyte. *V*_1_ = −750 mV, *V*_2_ = 50 mV. Effective potential applied to WE was +725 mV vs. Ag/AgCl. Tip approach and retraction rate both 1 μm s^–1^, which can be used to convert time to tip–substrate distance in the approach and withdraw segments of the plots.

In the hybrid SECCM-IRM configuration,^38,39,57^ an ITO-coated glass coverslip serves as an optically-transparent electrode. In this work, the coverslip was secured on the positioning stage, over the inverted microscope objective (Figure 1a). The top (ITO) side of the substrate, being conductive, was used as the cell WE, held at ground. It was connected to a current follower in order to record the substrate current, *I*_sub_. Initially, a ca. 500 nm diameter double-channel pipette was used as the tip, filled with a 50 mM KCl electrolyte solution. A bias potential *V*_2_ = 50 mV was applied between the two QRCEs, resulting in a continuous flow of ionic current between the two channels,^8,58^ denoted the tip current, *I*_tip_ (Figure 1a). This allowed indirect monitoring of the SECCM meniscus characteristics during all segments of the experiment (Figure 2), an advantage of the double-channel probe. It is noted that the reference potentials of the two QRCEs may slightly drift independently over time, resulting in *I*_tip_ measurements from subsequent landings to be offset by a certain value (related to the combined drift). Hence, the normalized tip current reported here is separately calculated for each approach-retraction (hop) by dividing *I*_tip_ by the value at the retracted (bulk) position, *I*_tip,bulk_. This accounts for potential drift and also for any small changes in the meniscus geometry between hops.

The QRCEs were additionally held at a bias potential *V*_1_ = −750 mV versus the ground (Figure 1a). When there is meniscus contact with the substrate, the effective applied potential^8^ to the substrate is then −(*V*_1_ + *V*_2_/2), or +725 mV versus the Ag/AgCl QRCEs in this case. The experimental procedure deviated from the typical SECCM scan, in the sense that the investigation was not targeting the activity of the sample surface. Rather, the focus was on the electrolyte meniscus behavior across multiple repeats of the same operation, each treated as a separate measurement.

During the course of the experiments, IRM images (SI, Figure S1 and Movie S1) are collected synchronously to the electrochemical data. Image contrast is achieved through local modification of the reflective condition, when the optical properties of the medium in the open space adjacent to ITO changes, e.g., from air to water when the meniscus lands.^59,60^ It is possible to detect the landing optically, depending on the characteristics of the optical instrument (light coherence, power, incidence, polarization, microscope objective, etc.), the nature of the semi-transparent substrate, and the size of the meniscus approaching the observation plane (the ITO–air interface).

For macroscopic interfaces, larger than the light wavelength, this situation corresponds to illuminating a planar multilayer stack. Fresnel equations describe how light is repeatedly reflected and refracted, leading to interference within such multilayer stack; and analysis of the reflected beam allows characterization of the stack dimension and optical properties.^61^ The strategy is commonly used in ellipsometric analysis of thin films, quantitative operando thin film (electrochemical) growth studies,^62−64^ or interferometric estimation of the size and position of objects, including of liquid droplets.^65^ The presence of a new planar air–water interface in the vicinity of the ITO substrate can then be detected with high sensitivity by evaluating changes in light reflectivity, over a distance range of the order of the micrometre. It follows that inspecting the light reflectivity changes, caused by an approaching SECCM meniscus, may provide a complementary means to assess its distance from the substrate.^66,67^

Objects smaller than the illumination wavelength can also be detected near the observation plane, as scattered light interferes with the beam reflected at the ITO–air interface.^68,69^ The range of detection is limited by the depth of field of the microscope objective. High magnification and high NA objectives, as used herein (63× magnification and NA = 1.4), provide high spatial resolution, but limit the depth of field to less than 1 μm — with an object being typically detected when it is within a few hundred nm away from the substrate in focus.^70,71^ In addition to any observable meniscus characteristics, formations (e.g., deposits) on, or close to, the WE are also readily distinguished, owing to the sensitivity of the interference effect to the local refractive index.^38,72,73^ This has been the focus of other studies, which also delve into the electrolyte medium.^74,75^

Here, the electrode wetted area, whose dimensions are comparable to the illumination wavelength used, is expected to show a mixed interferometric behavior, between reflectance at planar multilayer stack and interferometric scattering. Experimentally, the wetted area of the substrate appeared dark-contrasted with respect to the surrounding background area (Figure 1c), making the extent of the electrochemical cell directly observable *in situ*.^38^ As a first approximation, the variation in the optical contrast, between the meniscus-wetted region and the surrounding substrate area, can be explained by the variation in the reflection coefficient of the two interfaces probed: substrate–electrolyte and substrate–air, respectively. An estimate of the expected change is given by the Fresnel reflection coefficient equation, *R* = [(*n*_1_–*n*_2_)/(*n*_1_+*n*_2_)]^2^ with *n*_1_ the refractive index of the substrate and *n*_2_ that of the upper medium, electrolyte or air. For example, in the simplest case of comparing a glass–air (*n*_1_ = 1.46, *n*_2_ = 1) with a glass–water (*n*_2_ = 1.33) interface, one expects a ca. 94% reflectivity decay from 0.034 to 0.002, which explains the dark-contrasted meniscus. We note that the ITO coating employed in our experiments leads to less contrast than between the simple interfaces discussed above.

During a typical SECCM approach the probe was translated towards the surface until the tip current reached a set threshold (see description above), at which point it was kept stationary for 500 ms, before being retracted. The time point when the current threshold was reached was defined as the pipette landing and times are reported referenced to that point (set as zero). As an initial optical measure, the intensity value at the center of the wetted spot was normalized against a background value, outside the landing area. Measurements from a representative SECCM landing are shown in Figure 2, with both the normalized *I*_tip_ (see earlier definition, blue trace) and the IRM intensity traces (orange trace) reflecting the different segments of the operation. With the probe far from the surface, all electrochemical and optical signals had stable values. The optical intensity trace was the first to report the approaching pipette. It is optically detected several hundred nm away from landing: in Figure 1c-ii, it is detected as a disk brighter than the background (cf. Figure 1c-i) and then becoming darker than the background (Figure 1c-iii). The optical feedback (orange trace in Figure 2a, and discernible more clearly in the time-averaged trace, Figure 2b) suggests that the pipette is detected when approximately one tip diameter (500 nm; time ≈ −0.5 s) away from landing, the value begins to fluctuate due to periodic constructive and destructive interference involving the reflection of the approaching hanging cell (SI Movie S1).

**Figure 3:**
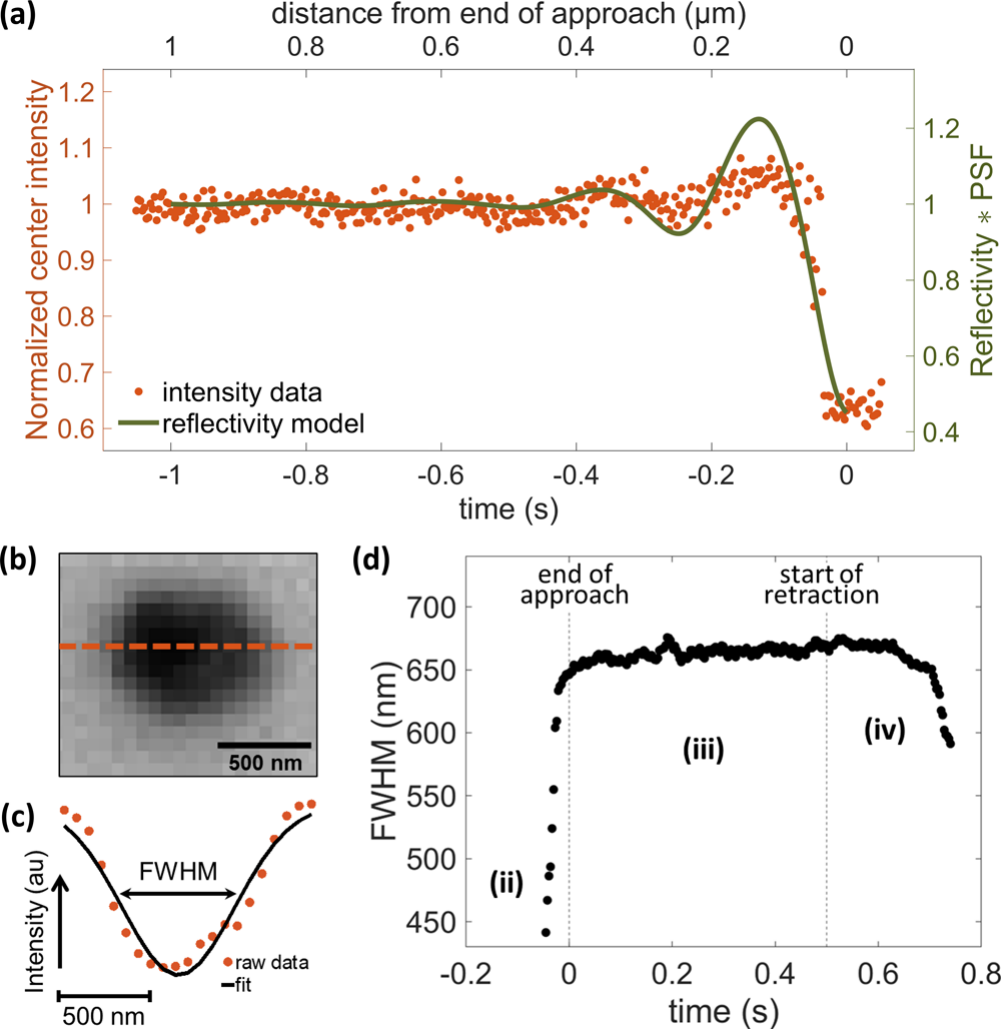
Quantitative optical analysis of meniscus behavior during approach and retraction. (a) Normalized pixel intensity at the center of the landing spot (orange points, left vertical axis) shown along with the simulated relative variation of reflected light (green trace, right vertical axis — considering the reflectivity of a planar interface and the PSF of the microscope). Time zero refers to the end of approach (synchronized optical intensity). The simulated variation of light is evaluated (see SI Figures S4–S5) for different substrate–object separation distances, which are then converted into time from the probe approach rate. (b) A typical IRM movie frame during meniscus contact with the surface, showing the position of the line profile (orange dotted line) used for analysis. (c) The intensity profile (orange points) and FWHM measure obtained from the Gaussian fit (black line). (d) FWHM — calculated in nm — from the fitted intensity line profiles of the IRM images, from the same experiment as Figure 1c and Figure 2, taken around the period that the pipette was held in meniscus contact with the surface. The FWHM values reported are averaged in time by a 9-sample window. Labels (ii)–(iv) correspond to the different stages highlighted in Figure 1. Mean FWHM and standard deviation, along with raw traces of 16 hops are displayed in the SI, Figure S6. The experiment used a ca. 500 nm tip diameter double-channel pipette with 50 mM KCl electrolyte. *V*_1_ = −750 mV, *V*_2_ = 50 mV. Effective potential applied to WE was +725 mV vs. Ag/AgCl. Tip approach and retraction rate both 1 μm s^–1^, which can be used to convert time to tip–substrate distance in the approach and withdraw segments of the plots.

When sufficiently close to the substrate (represented in Figure 1b-ii), the normalized current, *I*_tip_, then also deviated from its stable bulk value: it initially decreased (Figure 2b), indicative of a contraction of the meniscus volume and increased ionic resistance, before then rapidly increasing as the electrolyte wetted the surface. This pattern was supported by the IRM images (Figure 1c), in which the dark contrasted circular spot grew slightly over time: a detailed description of this phenomenon based on the spot size variation is presented below in Figure 3. During the stationary period with the meniscus in contact with the surface (Figure 1b-iii), *I*_tip_ remained generally stable. A small amplitude periodic oscillation emerged (Figure 2a), but is attributed to mechanical noise from the microscope camera (frequency analysis presented in SI Figure S2).

Upon pipette retraction, an increase in normalized *I*_tip_ was observed (Figure 2a), reflecting the meniscus being stretched (Figure 1b-iv), while the wetted area increased further slightly. At the point of tip detachment from the locally wetted surface, the current value rapidly returned to the level monitored before approaching (bulk value, Figure 2a). The optical intensity recovered as well, at a lesser value due to some electrolyte residue that remained on the surface. Intensity fluctuations were detected again, until they diminished, when the tip–substrate distance increased substantially.

In the absence of a redox compound in the solution, the substrate current *I*_sub_ (Figure 2a, purple trace) remained negligible and largely unchanged throughout the whole operation. A charging spike — near the wetting time and, occasionally, the detachment — was observed (Figure 2b). During meniscus contact and because of the applied bias to the substrate (effective +725 mV vs. Ag/AgCl), a different substrate current level developed, resulting from the water oxidation reaction. This change was mostly evident after time-domain averaging of the current–time series.

The optical intensity oscillations (Figure 2b, orange trace) and their sharp decrease close to meniscus contact can be rationalized, based on the Fresnel formalism of light reflection at a planar interface (for objects larger than the light wavelength). As discussed above, the difference in the reflection coefficient, between the cases of substrate–air and substrate–water interfaces, results in the observed image intensity transient (Figure 2, orange traces) and the wetted area contrast (e.g., in Figure 1c and in the SI, Figure S1 and Movie S1). The formalism also explains qualitatively — but not satisfactorily (see the improvement detailed below) — the oscillations of the optical intensity, when considering the multiple reflections at a substrate–air–water multilayer stack with varying air layer thickness (i.e., varying substrate–meniscus separation distance).

In principle, an estimate of the absolute substrate–meniscus separation may be reached from such formalism, especially when using polarized or coherent light sources, or a differential interference contrast system. The system studied here may, however, possess weak optical coherence, owing to, *inter alia*, the non-planarity of the meniscus–air interface, and the large substrate–meniscus separation. Such complication of optical incoherence can be taken into account when applying the Fresnel formalism, by averaging the (simulated) intensity of reflected light over a wide range of incidence angles.^61^ This provides an estimate of the amount of light reflected by the meniscus. In this respect, the pipette end can be treated as light emitting domain placed at a given distance from the substrate, which corresponds to the focal plane of the microscope objective. The amount of light collected by the objective by such a point source can then be evaluated from the knowledge of the axial point spread function (PSF) of the object for the microscope used. This is analogous to the localized-based fluorescence microscopy approaches.^76,77^

Among the various computing methods that have been proposed to predict the three-dimensional PSF of a microscope and evaluate the localization of a point source from the light collected by the microscope,^78,79^ we employ here the Gibson-Lanni model.^80,81^ It allows us to evaluate the light, reflected by the meniscus (point source) and reaching the objective, at discrete distances from the focal plane. The resulting simulated relative variation of reflected light, against the substrate–meniscus separation distance, is presented in Figure 3a (green trace), overlaid onto the raw experimental optical approach curve (orange points, also shown in Figure 2). The different steps of the calculation are described in the SI, Figures S4–S5. The simulated curve reproduces closely the experimental trends discussed previously, i.e., dampened oscillations over several hundreds of nanometres, and in particular the sharp decrease in reflected light when the water interface is less than 150 nm from the substrate. Despite undesirable noise in the optical intensity readout, there is good agreement of the simulated curve with the approach analyzed in Figure 3a and for the set of 16 approach curves in SI Figure S5. It suggests that the optical signal may be a usable proxy for the SECCM probe–substrate separation distance, and a valuable measure towards further SEPM instrument automation.

For example, one can use the onset of optical intensity oscillations to identify the near-contact region in its earliest stage, ca. 500 nm to 1 μm away from the substrate, as the oscillation amplitude increases with decreasing separation. Since the oscillations are caused by optical interference, they follow a period of λ/2 (here approximately 235 nm) and they are detectable earlier than any change to the meniscus geometry (see tip current *I*_tip_, Figure 2). The sharp decrease in intensity is another relevant indicator, with a linear decrease observed during the last 150 nm before contact (Figure 2b & 3a) — with the exception of the last ca. 10 nm which are difficult to analyze (due to meniscus distortion and close proximity of the pipette opening).

Further quantitative insight on the dynamics of the meniscus interaction with the substrate can be gained from analyzing a line profile across the center of the dark-contrasted spot, on the IRM images during meniscus contact (Figure 3b). The full width at half maximum (FWHM) of a Gaussian fit to the extracted intensity values (Figure 3c) is used to represent the apparent optical diameter of the wetted area. Although this apparent optical diameter is still convoluted with the PSF of the objective, it can be estimated according to super-localization principles, with sub-100 nm precision,^82^ providing higher accuracy in the evaluation of the wetted area size variation than standard binary threshold analysis. As with the raw intensity values, the same line profile can be used to indirectly study the meniscus in time, throughout the respective hop: before, during, and after contact with the substrate. Figure 3d reports on the FWHM — from the same pipette landing as the IRM images in Figure 1c and the traces in Figure 2 — converted in nm, by using the camera sensor pixel size and objective magnification. The measure is further averaged in time, with labels (ii)–(iv) corresponding to the hop stages shown in Figure 1b and 1c.

Just before contact, the images capture the hanging droplet from the pipette tip, and it is therefore possible to estimate the SECCM probe tip optical size by the measured FWHM. The intensity fluctuations during approach hindered analysis of all IRM snapshots; but for the last ca. 30 nm before making meniscus contact, the FWHM value was comparable to the expected tip diameter of ca. 500 nm. After a slight compression of the meniscus, identified from the tip current (see Figure 2), a jump to contact was then observed and the FWHM increased by ca. 30% to ca. 660 nm over 50 ms, as the landing spot was wetted fully. In the course of the stationary period (500 ms, Figure 3d-iii), the wetted area diameter increased marginally, particularly during the first 100 ms, here ca. 30 frames. The retraction step then followed (Figure 3d-iv), and a further FWHM increase was observed, reaching ca. 670 nm in the time the probe was retracted by ca. 100 nm. Subsequently, the wetted area shrank to less than ca. 620 nm in diameter (pipette retraction by ca. 250 nm), before meniscus detachment occurred. These changes in size of the IRM feature seen during pipette retraction reflect the SECCM meniscus geometry change, with the droplet stretching between the pipette and the substrate, affected by the wetting of the pipette wall, the ITO surface, and the solution surface tension.

The effect of substrate potential on the observed wetting was tested by performing an array of SECCM landing experiments and varying the bias potential *V*_1_ at each new approach, in intervals of 100 mV, between −750 and +750 mV — effective bias at the WE between +725 and −775 mV vs. Ag/AgCl. It is expected that the ITO substrate is stable and that no reactions take place within this potential window, when using a KCl solution only (see below for a modified electrolyte solution also used).^38,83^ The FWHM across 16 hops retained the same trend (SI Figure S6), suggesting that the meniscus wetting area remained relatively unaffected by the effective applied potential, within this potential value range.^38^ After the cell settles and throughout the stationary period, it shows significant stability, with the FWHM varying by 2.63%, on average (over the 16 landings, standard deviation of 0.3 percentage points).

The analysis exhibits some of the complementary information that this hybrid method can provide, that extend past the detection of phase formation and change within the miniature cell.^38,39^ The pipette tip diameter estimate — taken from its optical diameter, the FWHM value, just before contact — is expected to provide accurate measurement, once calibrated with corresponding electron microscopy imaging.^53^ This advancement would eliminate the need for measuring the tip opening *ex situ*, as long as the tip diameter is significantly larger than the diffraction limit (equal to λ/(2NA) ≈ 168 nm in this configuration). Even in the case of an optical feature comparable in size to the diffraction limit, relative changes (as in monitoring FWHM variation during the holding-landed segment of the 500 nm-diameter probe) are accurately captured, since the FWHM is a linear function of size. The time-evolution dynamics of the electrochemical cell dimensions is also evident during the experiment. Jointly with the electrochemical response, the IRM images allow one to easily diagnose problematic landings and to tweak the experimental protocol accordingly.

In order to obtain additional information on the dynamics of the miniature electrochemical cell, a variation on the pipette–meniscus–substrate system was explored. A redox-active species was added to the electrolyte solution, to seek a fast and clear response in the substrate current, *I*_sub_. The latter is useful for distinguishing between successful and failed landings and inconsistent meniscus wetting. A solution of 0.5 mM FcDM in 25 mM KCl was chosen, and a ca. 1.8 μm tip diameter double-channel pipette was preferred. The potential *V*_2_ between the two pipette channels was kept at 50 mV, while the QRCEs were now further biased at *V*_1_ = −400 mV, to be within the potential window for FcDM oxidation (WE held at an effective potential of approximately +375 mV vs. Ag/AgCl).

SECCM probe translation towards a homogenous at this scale^26^ sample (e.g., a clean, unmodified ITO-coated glass coverslip, but also a range of samples studied with SECCM) typically results in full wetting of the landing spot. Many factors play a role here, such as the physical properties of the substrate material, type of electrolyte solution,^43,45,84^ approach speed, final tip–sample separation, ambient conditions,^16,27^ and applied potential. Under these experimental conditions, the wetting was identified by the normalized *I*_tip_ shifting to a higher value (after its temporary decrease just before contact); *I*_sub_ suddenly deviating from zero to a finite value (non-wetting to wetting); and the IRM image features and analysis thereof.

**Figure 4:**
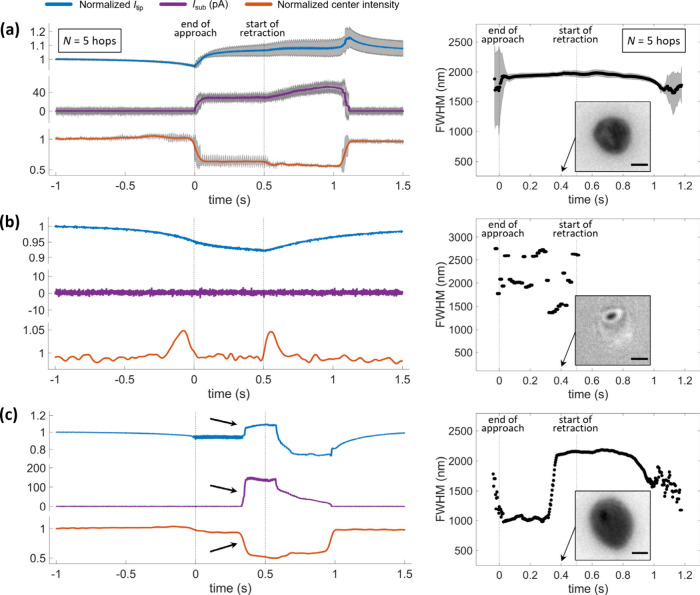
Left: Normalized tip current (blue), substrate current (purple), and normalized intensity at the center of the landing spot (orange) during three approach–retract scenarios: (a) successful approach, (b) non-wetting approach, and (c) approach with delayed wetting. Right: FWHM analysis of the same scenarios, around the period that the pipette was held stationary, and representative IRM snapshots at 0.4 s after end of approach. 5 individual, typical approaches were combined to produce the traces in (a), with the colored traces representing the respective mean and the grey shading showing ±1 standard deviation. All traces reported on the left are averaged in time by using Gaussian filter and a 25-sample window. Raw traces are displayed in the SI, Figure S7. Arrows in (c) mark the moment the meniscus wets the surface in a delayed wetting case. Scale bars represent 1 μm. The experiment used a ca. 1.8 μm tip diameter double-channel pipette with 0.5 mM FcDM, 25 mM KCl electrolyte. V_1_ = −400 mV, V_2_ = 50 mV. Effective potential applied to WE was +375 mV vs. Ag/AgCl. Tip approach rate 1 μm s^–1^; retraction rate 1.5 μm s^–1^.

A set of the above-mentioned traces, produced by analyzing 5 typical, successful approaches using the same pipette (ca. 1.8 μm tip diameter), is presented in Figure 4a. The normalized *I*_tip_ trace (mean and further averaged in time by using a Gaussian filter and a 25-sample window, in blue, and ±1 standard deviation, in grey) had a form similar to previous observations (Figure 2). Here, the wider pipette aperture and the larger tip–sample separation led to an *I*_tip_ with less pronounced transitions, across all approach stages. The normalized measure in Figure 4a reports on the pipette landing, with its value increasing during retraction and peaking close to the tip detachment point, where the droplet is at “full stretch”, enhancing ionic transport between the channels.

The addition of FcDM and the choice of the *V*_1_ bias gave rise to a substantial substrate current. The attained *I*_sub_ value remained stable during meniscus contact, limited by mass transport at the pipette–substrate gap. It increased slightly upon the start of pipette retraction, due to a change in the cell shape and associated mass transport effects; before dropping back to zero upon detachment. The optical recording (SI Figure S8 and Movie S2) helped to consolidate the information from the two distinct electrochemical signals and determine the wetting stage at which current is passed through the substrate. At the same time, it can be used as a sufficient measure by itself, to single out cases of no or irregular wetting (see the discussion below). In the right part of Figure 4a, an IRM image of the meniscus (during the stationary period in contact with the surface) and the FWHM analysis reflect the typical behavior observed in Figure 3 for the smaller pipette aperture. Interestingly, the gradual wetting dynamics reported in the optical signal are also mirrored in the *I*_tip_ and *I*_sub_ traces (current increase). These variations of the current are mostly attributed to the change in the meniscus shape during the first 100 ms of wetting.

An inappropriate choice of experimental parameters, such as the current threshold (*I*_tip_ in a double-channel or *I*_sub_ in single- or double-channel pipette SECCM) for halting the approach or the approach speed, can sometimes result in a failed meniscus landing. We investigated these circumstances by choosing a relatively low current threshold (i.e., small relative change allowed). Figure 4b shows an example of a false approach, but for which *I*_tip_ exceeded the specified threshold value, thus interrupting the probe’s approach. In such cases, the proximity was manifested with a dark-contrasted feature in the IRM image (Figure 4b, and SI Figure S9 and Movie S3) and a relative decrease in the intensity trace. The decrease was much smaller than observed in a successful approach (Figure 4a), once more showing that the SECCM droplet was hovering above the surface without contact. Here, signal oscillations due to mechanical vibrations hid this change, but the coupling between the pipette and substrate systems was evident after time averaging (raw traces displayed in SI Figure S7b): a period of low-frequency oscillations was mirrored before and after the high-frequency oscillations of the stationary period. However, the IRM images showed no wetting occurring, and no current was passed through the substrate. FWHM analysis returns incomplete and ambiguous results in this case, as demonstrated in Figure 4b.

In a different scenario, a delayed wetting of the landing spot took place, when an initially false approach evolves to full meniscus contact within the imposed stationary period. The event was documented with the appropriate transitions in the tip current and optical recordings (Figure 4c, with arrows marking the moment of wetting, and SI Figure S10 and Movie S4). The period before wetting was different from the previous case (Figure 4b), when no wetting occurred throughout. Here, the FWHM analysis revealed the meniscus, in close proximity to the substrate, to be more stable before the break-through wetting event. The substrate current transient further verified that only after wetting occurs — whether that may be immediate or delayed — was current recorded at the working electrode.

## Conclusions

In this work, a coupled SECCM-IRM instrument was utilized to image the meniscus-electrochemical cell during SECCM operation and measurement, and to analyze the meniscus status in detail and quantitatively. A double-channel pipette probe allowed for constant, indirect monitoring of the hanging cell geometry (via the tip current, *I*_tip_, between the two barrels), concurrently with observations of the electrochemical processes at the working electrode (via the substrate current, *I*_sub_, at the WE). *In situ* optical recordings visualized the working surface with high temporal and spatial resolution, tracking the extent of the electrochemical cell directly and throughout the experiment. A one-to-one correlation between electrochemical and optical signals was observed during the typical approach, measurement, and retraction segments of the SECCM scanning procedure in hopping mode. IRM images were used to identify the approach of the hanging meniscus in the last 500 nm from the substrate, and also the wetting occurrence and status, as demonstrated with scenarios of no, or delayed, wetting. In the case of the moderate potential range that was used here, the meniscus wetting on the WE surface was not found to be affected by the applied potential. However, we note that SECCM-IRM provides an excellent testbed for electrowetting experiments.^44^

The combined SECCM-IRM method advances the understanding of the *I*_tip_ behavior in the double-channel pipette configuration, by introducing additional markers and *operando* monitoring of the electrode–electrolyte interface. It is, of course, generally applicable to any SECCM configuration, as has been shown previously, in tracking phase formation within the meniscus.^32,38,39^ Analysis resulting from this work can also shed light to aspects of the typical SECCM experimental protocol that can be improved. Failed or abnormal landings are normally identified by *ex situ* sample characterization or analysis of the recorded data. Now, parameters such as the approach rate to the surface, or an additional holding period before acquiring the measurement can be easily evaluated and adjusted to fine-tune an experiment — also considering the variety of semi-transparent substrates that can be utilized, e.g., coatings of ITO, gold, platinum, etc., acting as WE — and avoid adverse effects, like the occurrence of solute precipitation at the probe end. Furthermore, IRM images can be valuable in characterizing the pipette aperture size, without the need of *ex situ* electron microscopy. The immediate optical feedback offers an alternative method for probe positioning (i.e., by an optical approach curve), backed by additional automation features (e.g., machine vision). Finally, and beyond advising on practical considerations for the experiment, SECCM-IRM appears as a prime tool for collecting multimodal statistical information on the observed system, unravelling mechanistic details pertinent to surface adhesion, (electro)wetting, droplet deformation, and the behavior at liquid–liquid interfaces.

## Data Availability

Data will be made available on reasonable request.

## Supporting Information Available

Frequency analysis of an exemplary tip current signal, details of the optical simulation methods, and supplementary figures (image filmstrips and raw electrochemical and optical signals) to support the findings presented in this work (PDF)
Movie S1, optical recording of a single SECCM approach and withdrawal (hop), analyzed in Figures 1–3 and S1 (AVI)
Movie S2, optical recording of a single successful SECCM approach and withdrawal (hop), analyzed in Figures 4a and S8 (AVI)
Movie S3, optical recording of a single non-wetting SECCM approach and withdrawal (hop), analyzed in Figures 4b and S9 (AVI)
Movie S4, optical recording of a single SECCM approach and withdrawal (hop) with delayed wetting, analyzed in Figures 4c and S10 (AVI)

## Supplementary Material

ec4c00029_si_001.pdf

ec4c00029_si_002.avi

ec4c00029_si_003.avi

ec4c00029_si_004.avi

ec4c00029_si_005.avi
