# Supplementary material for: Operando Electrochemical and Optical Characterization of the Meniscus of Scanning Electrochemical Cell Microscopy (SECCM) Probes
Source: ACS Electrochem. 2024 Oct 7;1(2):153–63. doi: 10.1021/acselectrochem.4c00029 (PMC11808645; doi:10.1021/acselectrochem.4c00029)
Supplement: Supplementary file 1 — ec4c00029_si_001.pdf [file ec4c00029_si_001.pdf]

Supporting Information

Operando electrochemical and optical characterization of the meniscus of scanning electrochemical cell microscopy (SECCM) probes

Dimitrios Valavanis,[a] Paolo Ciocci,[b,c] Ian J. McPherson,[a,d] Gabriel N. Meloni,[a,e] Jean-François Lemineur,[b] Frédéric Kanoufi, [b*] and Patrick R. Unwin [a*]

[a] Department of Chemistry, University of Warwick, Coventry CV4 7AL, United Kingdom
[b] Université Paris Cité, ITODYS, CNRS, F-75013 Paris, France
[c] Institute of Catalysis Research and Technology, Karlsruhe Institute of Technology, 76344 Eggenstein-Leopoldshafen, Germany
[d] Department of Chemistry, Loughborough University, Loughborough LE11 3TU, UK
[e] Institute of Chemistry, Department of Fundamental Chemistry, University of São Paulo, 05508-000 São Paulo, SP, Brazil

* Corresponding authors
Frédéric Kanoufi: frederic.kanoufi@u-paris.fr
Patrick Unwin: p.r.unwin@warwick.ac.uk

The Supporting Information contains additional figures and the captions for the accompanying movies (optical recordings), to support the findings presented in the main article.

Additional figures

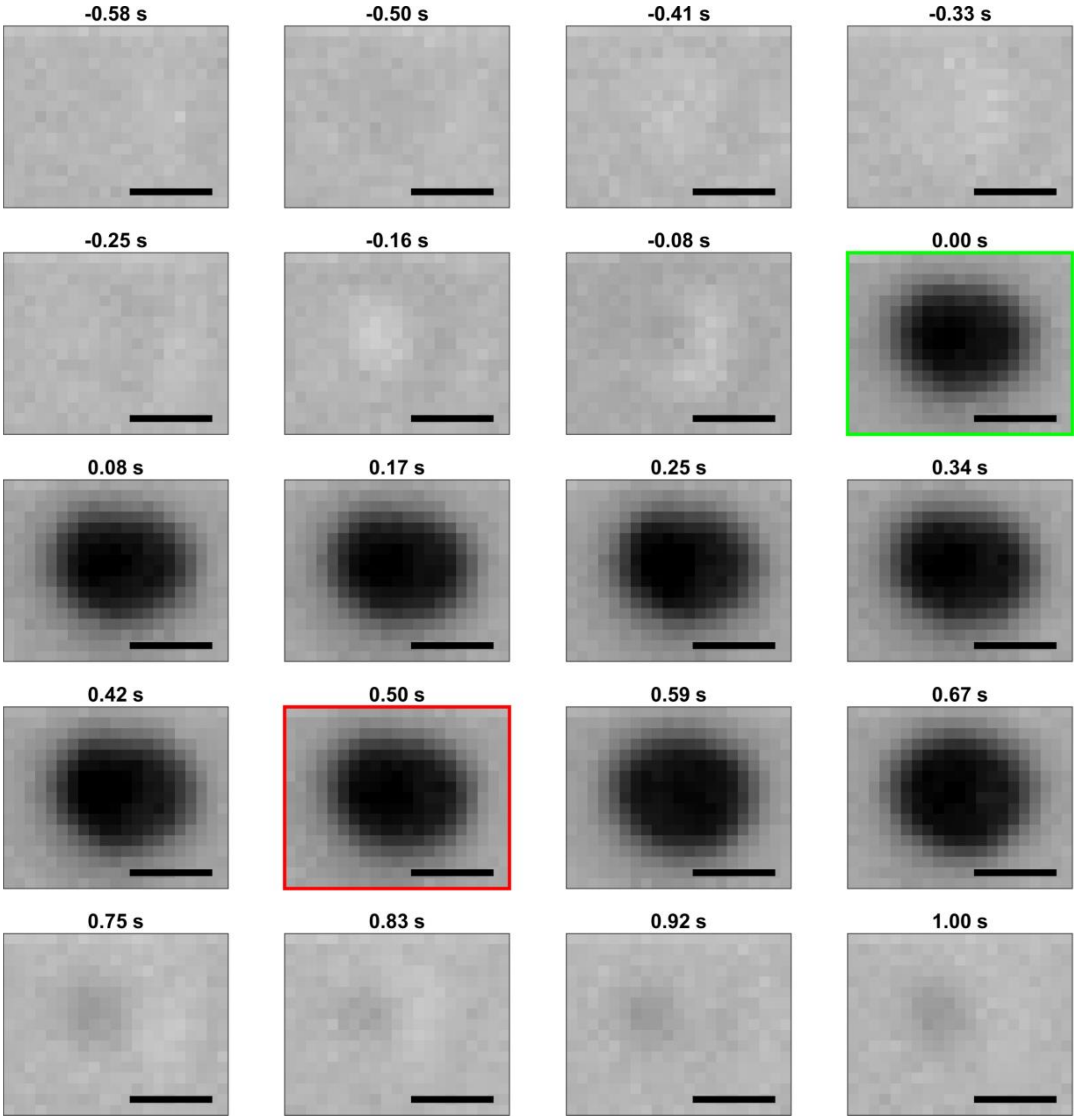

Figure S1 IRM image filmstrip from a single SECCM approach and withdrawal (hop), analyzed in Figures 1–3 of the main text. Each image represents the average over three consecutive frames. Images at the end of approach (0 s) and start of retraction (0.5 s) are highlighted with green and red border, respectively. Scale bars represent 500 nm. The experiment used a ca. 500 nm tip diameter double-channel pipette with 50 mM KCl electrolyte. V_1 = −750 mV, V_2 = 50 mV. Effective potential applied to WE was +725 mV vs. Ag/AgCl. Tip approach and retraction rate both 1 µm s^{-1}.

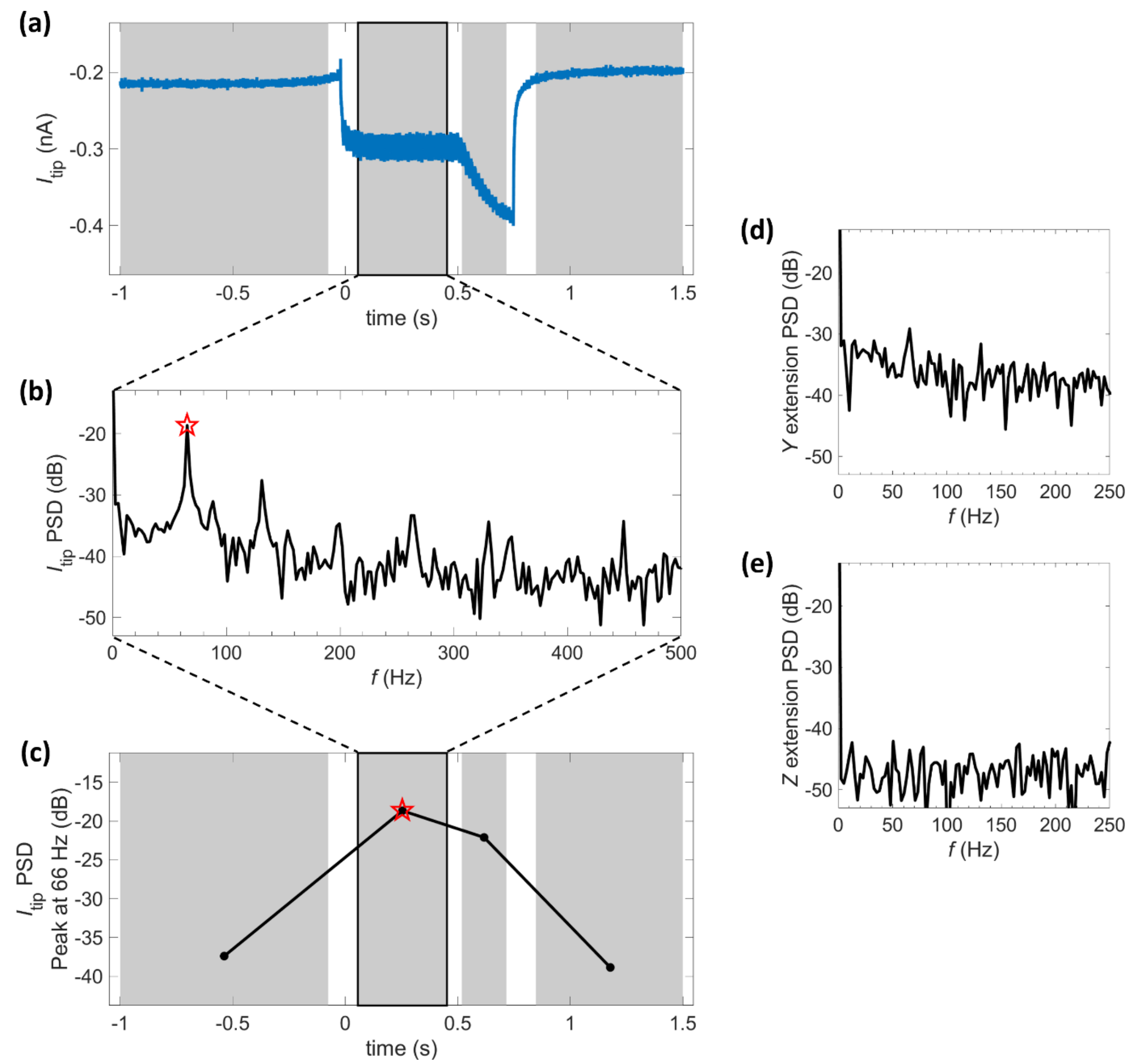

Figure S2 Frequency analysis of recordings from the exemplary SECCM hop analyzed in Figures 1–3 of the main text. **(a)** Tip current (blue) for a single complete SECCM hop. The trace shows raw (not normalized) I_{tip} values of the Figure 2a trace. Four sections (shaded with grey) were separately analyzed for the signal's frequency components: during approach and before meniscus contact with the surface; stationary during contact; during retraction and while still in contact; and during retraction after meniscus detachment. **(b)** Power spectral density (PSD) of the tip current, I_{tip}, during the stationary phase (approx. +0.05 to +0.45 s), while in meniscus contact with the surface. The red star denotes the primary peak value at 66 Hz, attributed to mechanical noise (see main discussion). **(c)** I_{tip} PSD value, at the frequency of 66 Hz, at each of the four sections. The red star denotes the same value as in panel (**b**). The frequency component at 66 Hz is highest during the second section (stationary and in contact), dampened during the third section (retraction and while still in contact), and minimal when not in contact. **(d-e)** PSD of the *Y* and *Z* piezoelectric positioner extension during the same stationary period as (**b**), revealing significantly lower, or indiscernible, frequency component

at 66 Hz. The experiment used a ca. 500 nm tip diameter double-channel pipette with 50 mM KCl electrolyte. V_1 = −750 mV, V_2 = 50 mV. Effective potential applied to WE was +725 mV vs. Ag/AgCl. Tip approach and retraction rate both 1 µm s^{-1}.

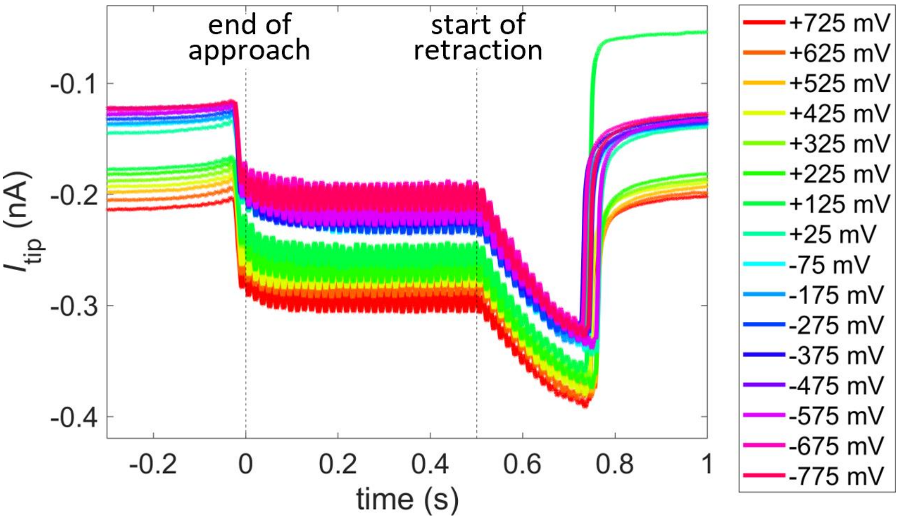

Figure S3 Raw I_{tip} traces across 16 hops with varied applied V_1 potential. The experiment used a ca. 500 nm tip diameter double-channel pipette with 50 mM KCl electrolyte. V_1 varied between −750 and +750 mV, V_2 = 50 mV. Effective potential applied to WE between +725 and −775 mV vs. Ag/AgCl. Tip approach and retraction rate both 1 µm s^{-1}.

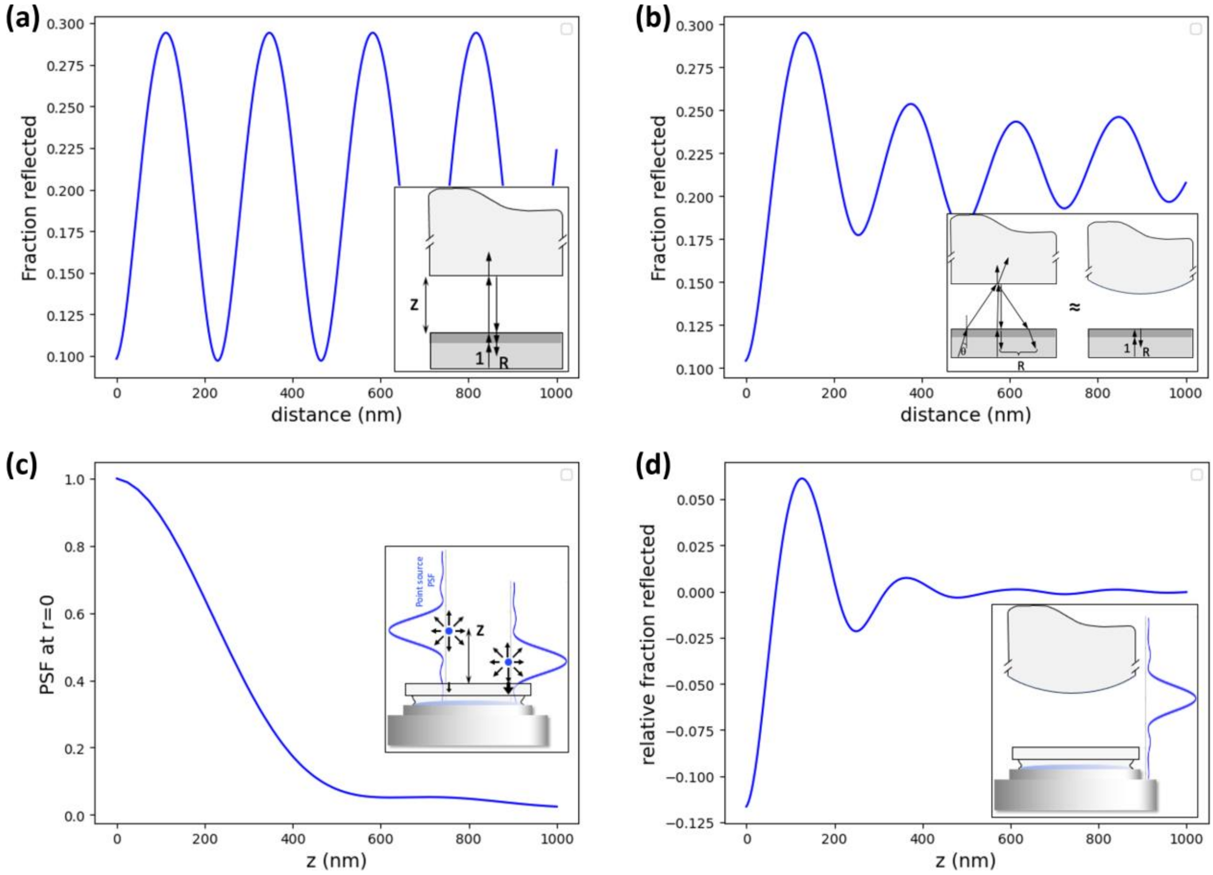

Figure S4 (a-b) Fresnel simulated fraction of λ = 470 nm light reflected by an ITO-coated coverslip (refractive index n_{ITO} = 1.95 + 0.005j, thickness 70 nm), illuminated under **(a)** normal incidence or **(b)** averaged over angles of incidence from 0 to 45° (angle for total internal reflection for n_{glass} = 1.46). **(c)** Point spread function along the axial axis for a point source located at different separation z distance from the focus plane (situated at z = 0). Parameters used: glass coverslip n_{glass} = 1.46, 63× oil-immersion microscope objective with NA = 1.4. **(d)** Product of the Fresnel simulated reflected light — in (**b**) but centered around its infinite distance asymptote — by the PSF (**c**). The different models employed are schematically described within each figure.

The averaged reflected light intensity in Figure S4a & b is obtained from transfer matrix method as provided in the transfer-matrix method optics package for Python (https://pythonhosted.org/tmm/tmm.html last accessed 2024-06-17), developed by Steven Byrnes according to details given in ref. 61 of the main article.

The PSF estimate for Figure S4c uses the Gibson-Lanni model as computed for a glass coverslip using the implementation for Python proposed by Kyle M. Douglass (refs. 80–81 of the main article).

For consistency between the Fresnel and PSF models, the simulated optical approach curve shown in Figure 3a and Figure S5 was computed for a glass coverslip (n_{glass} = 1.46), neglecting the influence of the ITO layer (not taken into account in the PSF model).

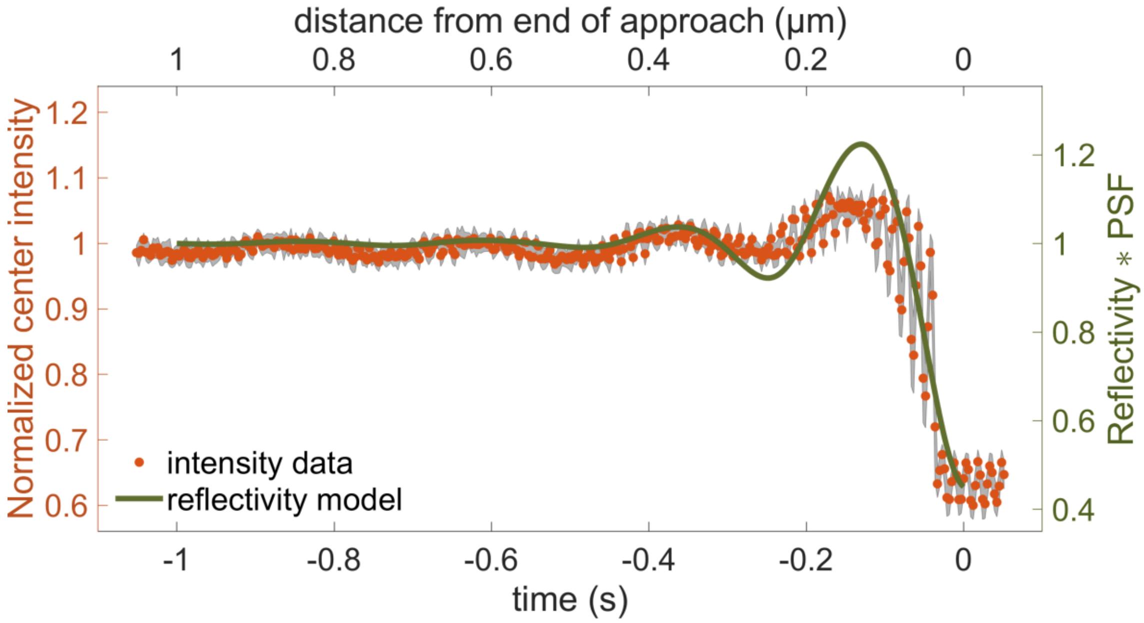

Figure S5 Mean (orange points) and ±1 standard deviation (grey shading) of the raw optical intensity variation across 16 hops with varied applied V_1 potential, compared with the Fresnel simulated optical approach curve (green trace; see also Figure 3a and Figure S4). The experiment used a ca. 500 nm tip diameter double-channel pipette with 50 mM KCl electrolyte. $V_1 = -750$ mV, $V_2 = 50$ mV. Effective potential applied to WE was +725 mV vs. Ag/AgCl. Tip approach and retraction rate both 1 μm s^{-1}.

The normalized reflectivity in the right vertical axis of Figure S5 (green trace) is evaluated from the simulated values in Figure S4b and S4d. The relative reflected intensity, ΔR (computed in Figure S4d), allows, with the simulated asymptotic fraction of reflected intensity, R_{lim} (evaluated from Figure S4b, $R_{lim} \approx 0.224$), to estimate the corrected fraction of reflected light considering all computed effects: $R_{corr} = R_{lim} + \Delta R$ from which the normalized reflectivity $R_{norm} = R_{corr}/R_{lim} = 1 + \Delta R/R_{lim}$.

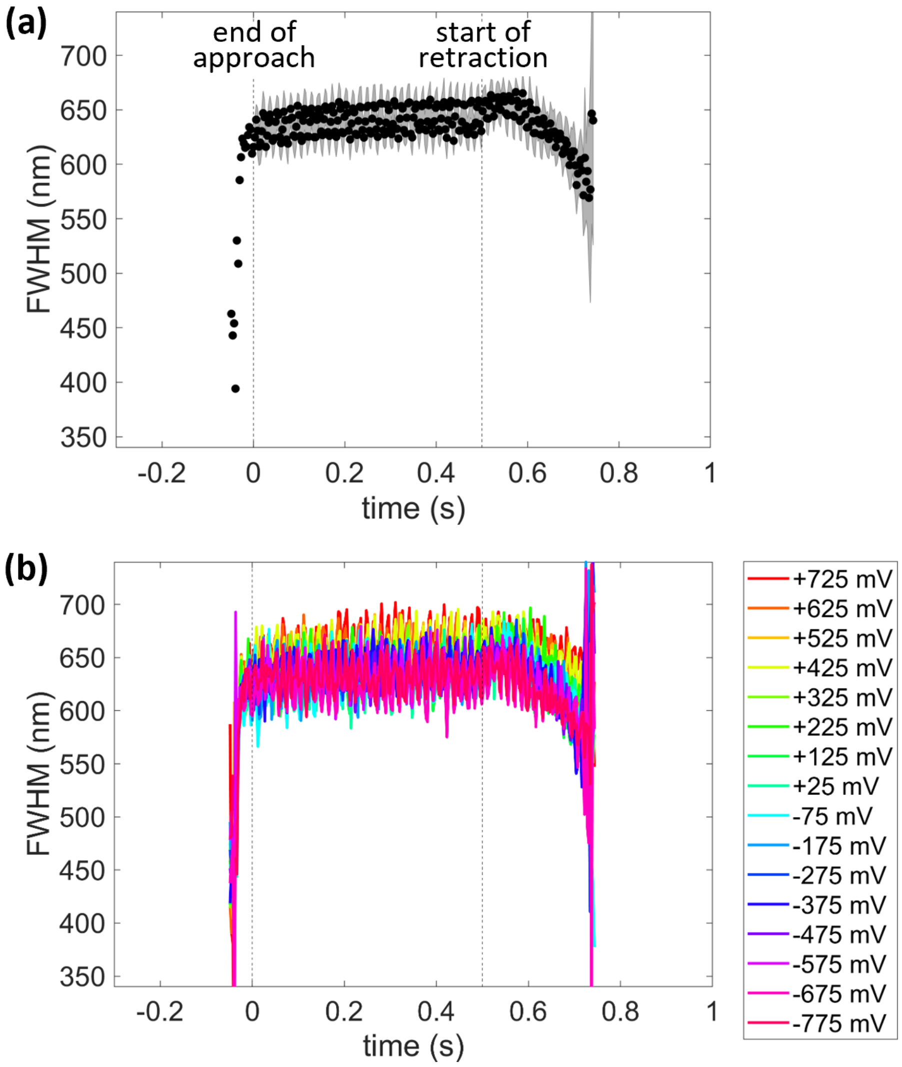

Figure S6 (a) Mean (black points) and ±1 standard deviation (grey shading) of the meniscus FWHM across 16 hops with varied applied V_1 potential. **(b)** Raw FWHM traces of the same experiment. The experiment used a ca. 500 nm tip diameter double-channel pipette with 50 mM KCl electrolyte. V_1 varied between −750 and +750 mV, V_2 = 50 mV. Effective potential applied to WE between +725 and −775 mV vs. Ag/AgCl. Tip approach and retraction rate both 1 μm s^{-1}.

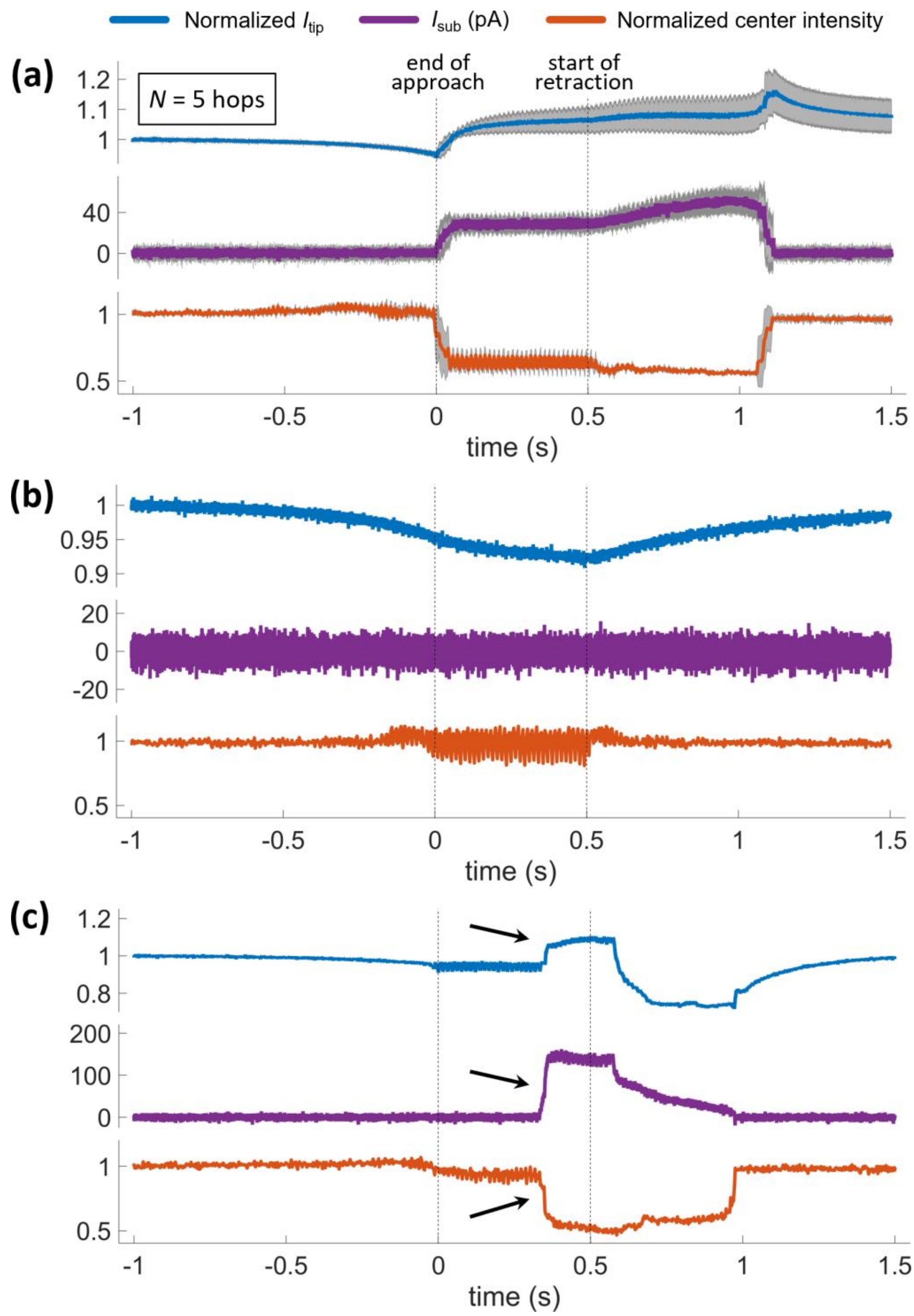

Figure S7 Raw (no time averaging) traces of Figure 4. Normalized tip current (blue), substrate current (purple), and normalized intensity at the center of the landing spot (orange) during three approach-retract scenarios: **(a)** successful approach, **(b)** non-wetting approach, and **(c)** approach with delayed wetting. The experiment used a ca. 1.8 μm tip diameter double-channel pipette with 0.5 mM FcDM, 25 mM KCl electrolyte. V_1 = −400 mV, V_2 = 50 mV. Effective potential applied to WE was +375 mV vs. Ag/AgCl. Tip approach rate 1 μm s^{-1}; retraction rate 1.5 μm s^{-1}.

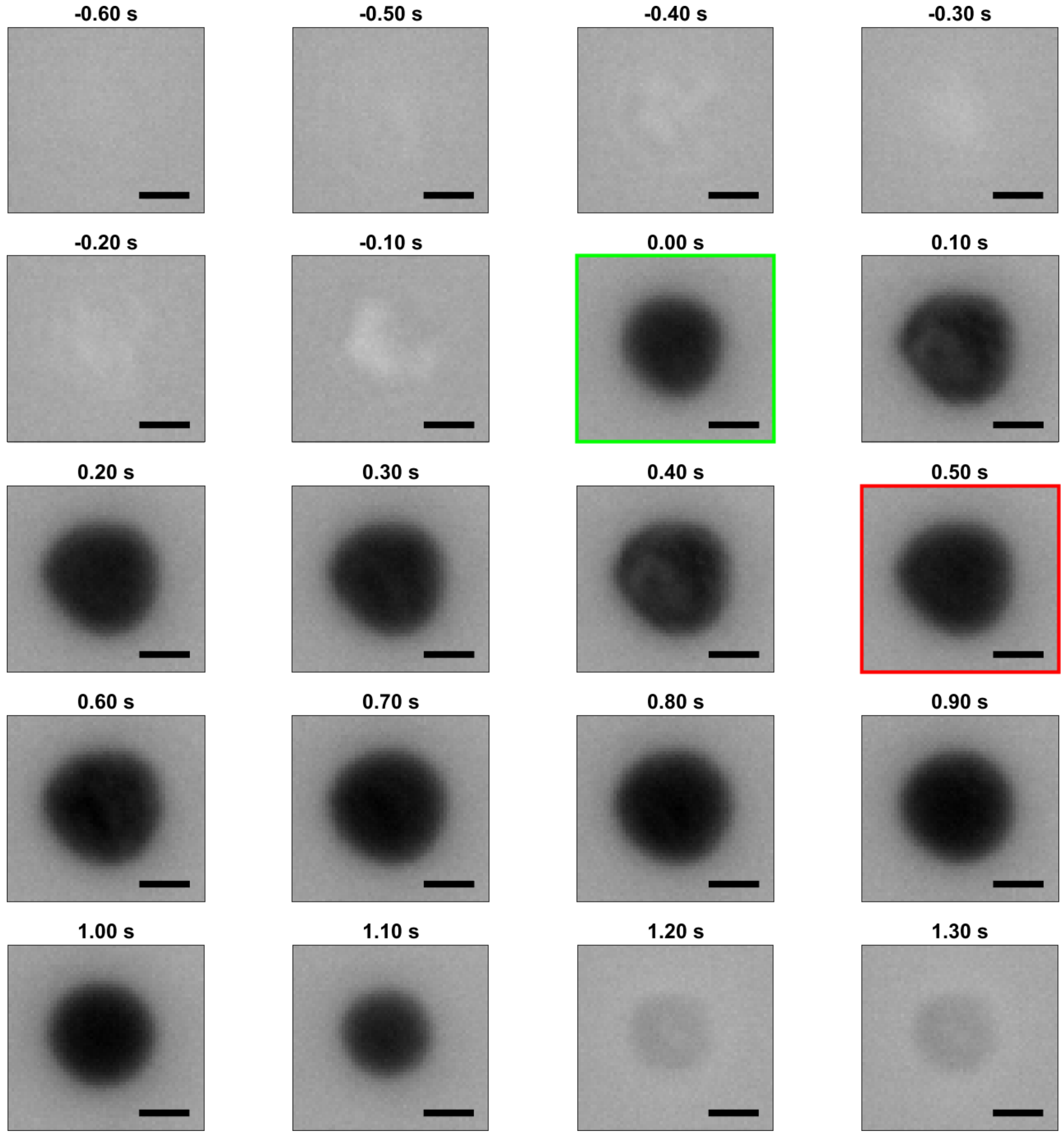

Figure S8 IRM image filmstrip from a single successful SECCM approach and withdrawal (hop), analyzed in Figure 4a of the main text. Each image represents the average over three consecutive frames. Images at the end of approach (0 s) and start of retraction (0.5 s) are highlighted with green and red border, respectively. Scale bars represent 1 µm. The experiment used a ca. 1.8 µm tip diameter double-channel pipette with 0.5 mM FcDM, 25 mM KCl electrolyte. V_1 = −400 mV, V_2 = 50 mV. Effective potential applied to WE was +375 mV vs. Ag/AgCl. Tip approach rate 1 µm s^{-1}; retraction rate 1.5 µm s^{-1}.

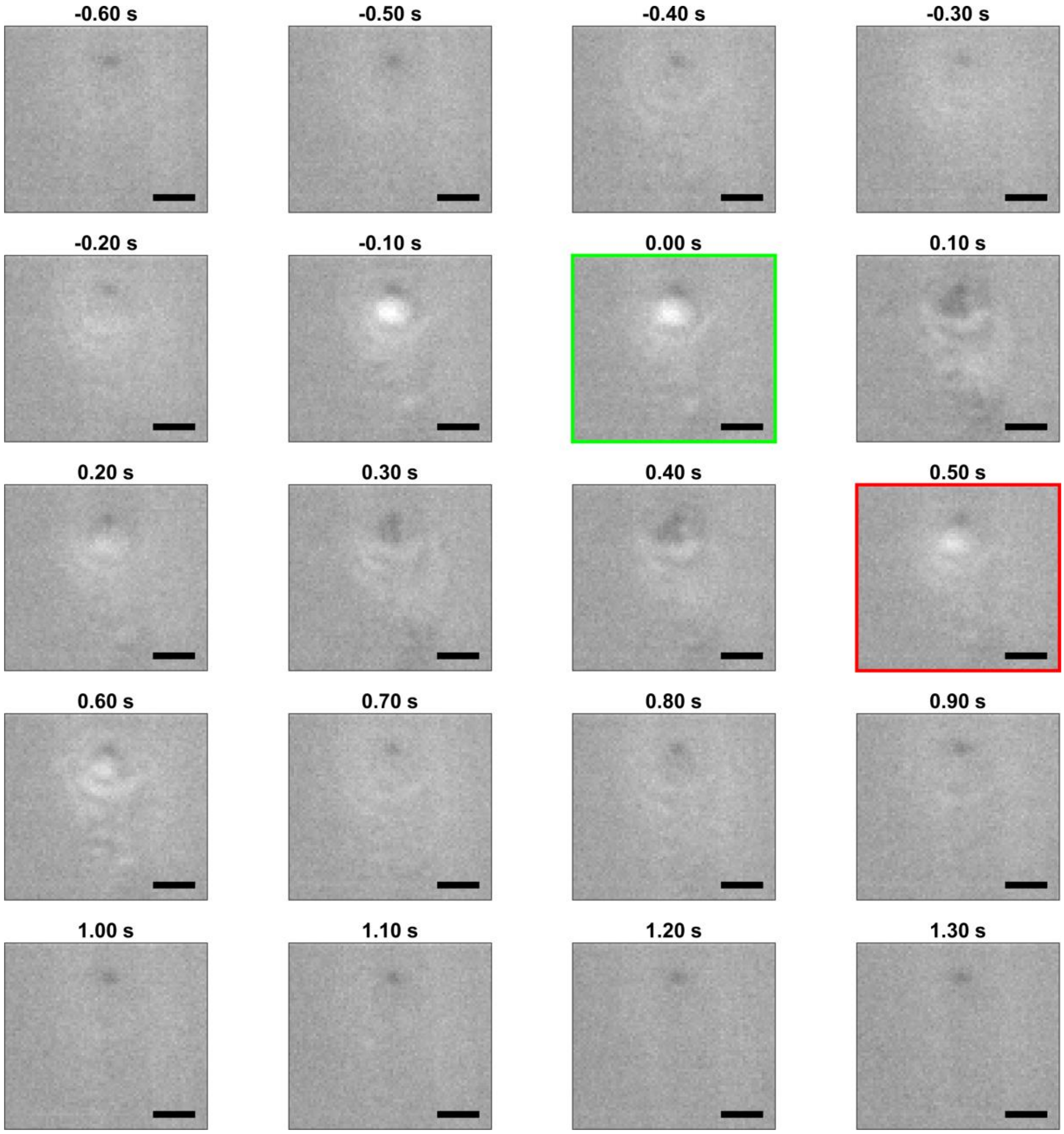

Figure S9 IRM image filmstrip from a single non-wetting SECCM approach and withdrawal (hop), analyzed in Figure 4b of the main text. Each image represents the average over three consecutive frames. Images at the end of approach (0 s) and start of retraction (0.5 s) are highlighted with green and red border, respectively. Scale bars represent 1 µm. The experiment used a ca. 1.8 µm tip diameter double-channel pipette with 0.5 mM FcDM, 25 mM KCl electrolyte. V_1 = −400 mV, V_2 = 50 mV. Effective potential applied to WE was +375 mV vs. Ag/AgCl. Tip approach rate 1 µm s^{-1}; retraction rate 1.5 µm s^{-1}.

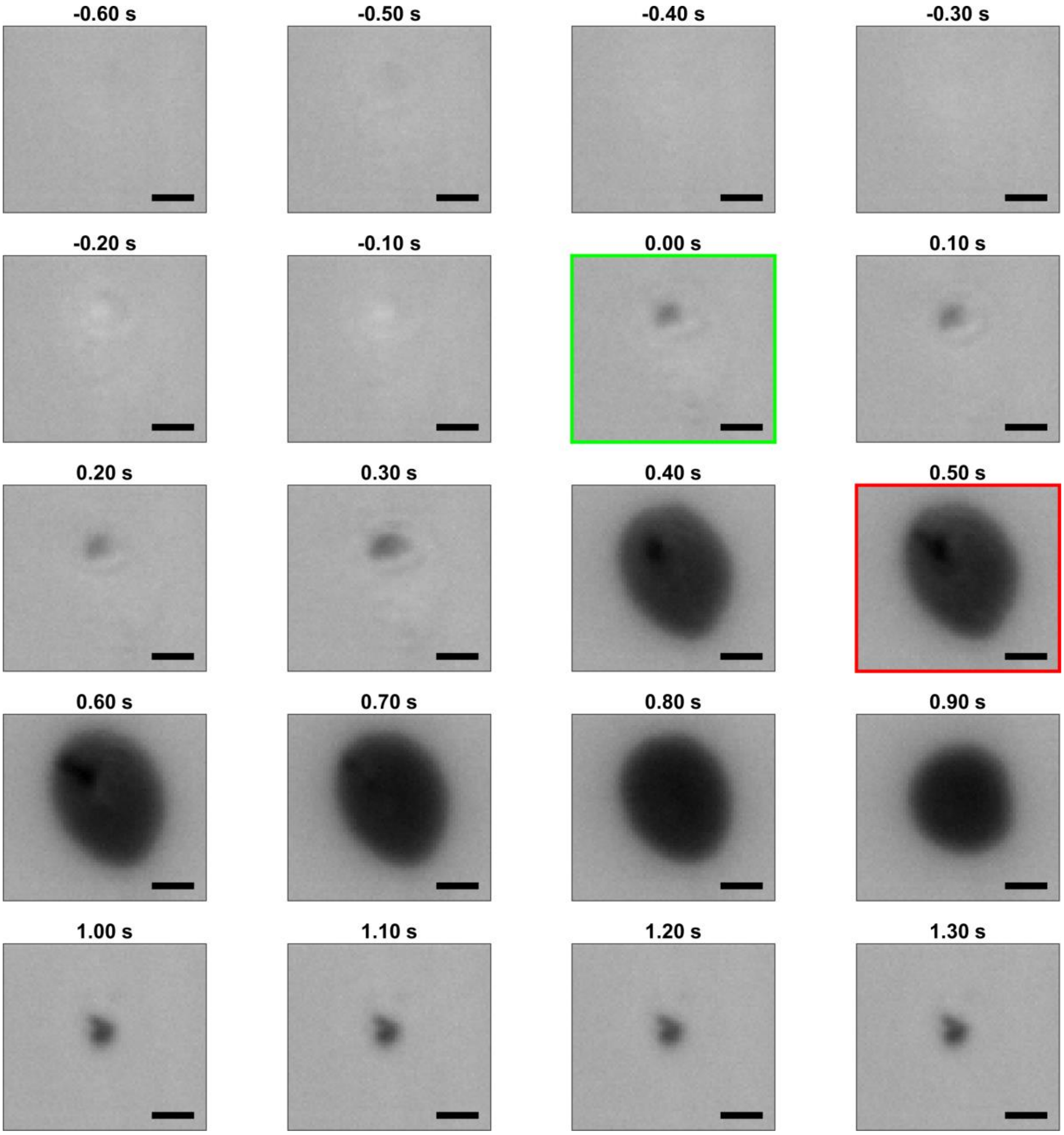

Figure S10 IRM image filmstrip from a single SECCM approach and withdrawal (hop) with delayed wetting, analyzed in Figure 4c of the main text. Each image represents the average over three consecutive frames. Images at the end of approach (0 s) and start of retraction (0.5 s) are highlighted with green and red border, respectively. Scale bars represent 1 µm. The experiment used a ca. 1.8 µm tip diameter double-channel pipette with 0.5 mM FcDM, 25 mM KCl electrolyte. V_1 = −400 mV, V_2 = 50 mV. Effective potential applied to WE was +375 mV vs. Ag/AgCl. Tip approach rate 1 µm s^{-1}; retraction rate 1.5 µm s^{-1}.

Movie captions

Movie S1 Optical recording of a single SECCM approach and withdrawal (hop), analyzed in Figures 1–3 and S1. The movie covers a period of ca. 1.6 s around the stationary (landed) period. The experiment used a ca. 500 nm tip diameter double-channel pipette with 50 mM KCl electrolyte. V_1 = −750 mV, V_2 = 50 mV. Effective potential applied to WE was +725 mV vs. Ag/AgCl. Tip approach and retraction rate both 1 µm s^{-1}.

Movie S2 Optical recording of a single successful SECCM approach and withdrawal (hop), analyzed in Figures 4a and S8. The movie covers a period of ca. 1.9 s around the stationary (landed) period. The experiment used a ca. 1.8 µm tip diameter double-channel pipette with 0.5 mM FcDM, 25 mM KCl electrolyte. V_1 = −400 mV, V_2 = 50 mV. Effective potential applied to WE was +375 mV vs. Ag/AgCl. Tip approach rate 1 µm s^{-1}; retraction rate 1.5 µm s^{-1}.

Movie S3 Optical recording of a single non-wetting SECCM approach and withdrawal (hop), analyzed in Figures 4b and S9. The movie covers a period of ca. 1.9 s around the stationary (landed) period. The experiment used a ca. 1.8 µm tip diameter double-channel pipette with 0.5 mM FcDM, 25 mM KCl electrolyte. V_1 = −400 mV, V_2 = 50 mV. Effective potential applied to WE was +375 mV vs. Ag/AgCl. Tip approach rate 1 µm s^{-1}; retraction rate 1.5 µm s^{-1}.

Movie S4 Optical recording of a single SECCM approach and withdrawal (hop) with delayed wetting, analyzed in Figures 4c and S10. The movie covers a period of ca. 1.9 s around the stationary (landed) period. The experiment used a ca. 1.8 µm tip diameter double-channel pipette with 0.5 mM FcDM, 25 mM KCl electrolyte. V_1 = −400 mV, V_2 = 50 mV. Effective potential applied to WE was +375 mV vs. Ag/AgCl. Tip approach rate 1 µm s^{-1}; retraction rate 1.5 µm s^{-1}.
